# Association between inflammatory bowel disease and atrial fibrillation: A systematic review and *meta*-analysis

**DOI:** 10.1016/j.ijcha.2024.101456

**Published:** 2024-07-25

**Authors:** Aman Goyal, Hritvik Jain, Surabhi Maheshwari, Jyoti Jain, Ramez M. Odat, Humza Saeed, Mohamed Daoud, Gauranga Mahalwar, Kamna Bansal

**Affiliations:** a Department of Internal Medicine, Seth GS Medical College and KEM Hospital, Mumbai, India; b Department of Internal Medicine, All India Institute of Medical Sciences (AIIMS), Jodhpur, India; c Department of Internal Medicine, University of Alabama, Montgomery, AL, USA; d Faculty of Medicine, Jordan University of Science and Technology, Irbid, Jordan; e Department of Internal Medicine, Rawalpindi Medical University (RMU), Rawalpindi, Pakistan; f Bogomolets National Medical University, Kyiv, Ukraine; g Department of Internal Medicine, Cleveland Clinic Foundation, Cleveland, OH, USA; h Department of Family and Community Medicine, Baylor College of Medicine, Houston, TX, USA

**Keywords:** Atrial fibrillation, Inflammatory bowel disease, Risk, Meta-analysis

## Abstract

**Background:**

Inflammatory bowel disease (IBD), including Crohn’s disease (CD) and ulcerative colitis (UC), is a prevalent condition associated with chronic noninfectious inflammation of the gastrointestinal tract. It has been hypothesized that chronic inflammation can predispose patients to atrial fibrillation (AF), however, no clear evidence exists to support this.

**Methods:**

A systematic literature search was conducted using major databases aimed at studies focusing on AF development in patients with IBD. Further subgroup analyses were performed for ulcerative colitis (UC) and crohn’s disease (CD). Risk ratios (RR) with their corresponding 95 % confidence intervals (CI) were pooled using a random-effects model in the Review Manager Software. Statistical significance was set at p < 0.05.

**Results:**

Seven studies with 88,893,407 patients were included (1,002,719 and 87, 890, 688 patients in the IBD and non-IBD groups, respectively). IBD patients were at an increased risk of developing AF [RR: 1.52; 95 % CI: 1.19–1.95; p = 0.0009] compared to the non-IBD group. In subgroup analyses, patients with UC were at an increased risk of developing AF [RR: 1.29; 95 % CI: 1.08–1.53; p = 0.004], as were CD patients [RR: 1.30; 95 % CI: 1.07–1.58; p = 0.008] compared to the non-UC and non-CD groups, respectively.

**Conclusion:**

Patients with IBD are at nearly 1.5 times the risk of developing AF compared to the non-IBD population. Our *meta*-analysis was limited by heterogeneity among the studies, highlighting the importance of further large-scale prospective studies to establish more robust evidence.

## Graphical abstract

**Figure:**
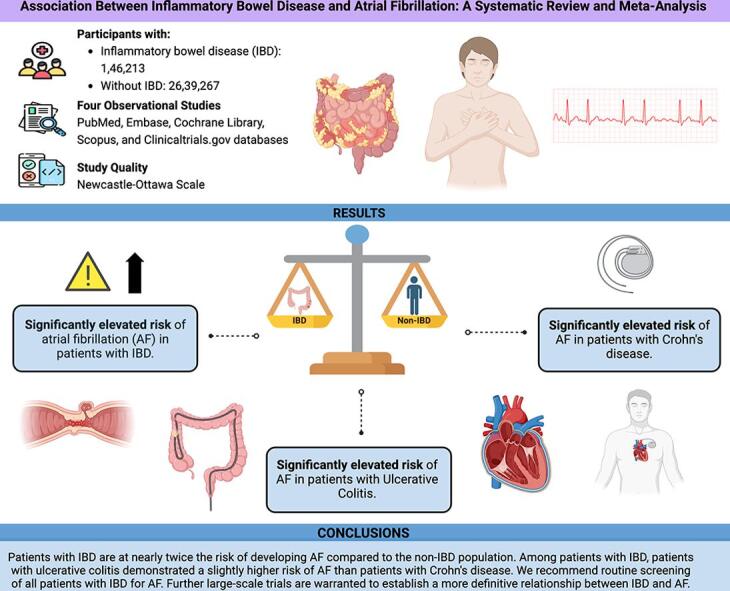

## 1 Introduction

Inflammatory bowel disease (IBD), comprising ulcerative colitis (UC) and Crohn’s disease (CD), is a prevalent clinical condition, with over 240 cases per 100,000 people in 2019 in the United States [1]. IBD is a waxing and waning condition characterized by episodes of inflammation in the gastrointestinal tract, caused by an abnormal response to the gut microflora [2]. Recent investigations have demonstrated an increased risk of cardiovascular diseases in patients with IBD due to the common interplay of risk factors such as obesity, diabetes mellitus, smoking, and diet [3]. IBD has been linked to an increased risk of coronary artery disease, peripheral arterial disease, stroke, deep vein thrombosis, and pulmonary embolism [4].

Interestingly, IBD has also been hypothesized to increase the risk of atrial fibrillation (AF), the most commonly encountered cardiac arrhythmia in clinical practice. The potential mechanistic link may involve systemic inflammation, autonomic dysfunction, and oxidative stress associated with IBD, as well as shared risk factors such as age, obesity and hypertension between the two conditions. Medications used to treat IBD may also play a role in the development of AF in these patients [5]. This hypothesis also stems from the fact that inflammation can act as a trigger for AF [6], [7].

The relationship between IBD and AF is not well-established, with inconsistent data on the association between the two conditions. This highlights the need for a comprehensive *meta*-analysis to systematically evaluate the risk of AF in patients with IBD compared to the general population. Hence, we conducted a *meta*-analysis to provide the most comprehensive pooled analysis of existing data on the risk of AF in patients with IBD.

## 2 Materials and Methods

This systematic review and *meta*-analysis were reported in line with the guidelines laid by the Preferred Reporting Items for Systematic Review and Meta-Analysis (PRISMA) guidelines [8]. We registered the pre-specified study protocol in the International PROSPERO registry under CRD42024534122.

### 2.1 Search strategy and eligibility criteria

A comprehensive literature searches of major electronic databases, such as Medline (via PubMed), Embase, Cochrane Library, Scopus, and the International Registry of Clinical Trials (https://www.clinicaltrials.gov), was performed from inception to March 2024. The search strategy for this *meta*-analysis was a combination of keywords and predefined Medical Subject Headings (MeSH) such as “inflammatory bowel disease”, “ulcerative colitis”, “Crohn’s disease”, “atrial fibrillation”, “auricular fibrillation”, “persistent atrial fibrillation”, and “paroxysmal atrial fibrillation”. We used the Boolean operators “AND” and “OR” to create the search strategy. The search strategy was modified according to the requirements of each database, as shown in Supplementary Table S1. The search was aimed at retrieving records that adhered to the following criteria: (i) Randomized controlled trials (RCTs) or observational studies, (ii) IBD patients in one group, (iii) non-IBD patients as controls, and (iv) assessment of the development of AF. Abstracts without published full texts, review articles, case reports, case series, editorials, correspondences, and viewpoint publications were excluded. Subsequently, duplicates were removed from all records using the EndNote Reference Library X7 software (Clarivate Analytics, Philadelphia, USA).

### 2.2 Data extraction and quality assessment

The following data were extracted from each study: author name, publication year, study design, country, number of participants, inclusion criteria, age group, male sex, diabetes mellitus, hypertension, dyslipidemia, and follow-up duration. Two investigators (H.J. and L.F.) extracted the data in an Excel spreadsheet, and a third investigator (A.G.) was consulted to resolve any discrepancies. Quality assessment of all observational studies was conducted using the Newcastle-Ottawa Scale (NOS) [9].

### 2.3 Data synthesis

All statistical analyses pertaining to this review were performed using Review Manager Software (version 5.4). We pooled dichotomous outcomes using risk ratios (RR) and their corresponding 95 % confidence intervals using the Mantel-Haenszel random-effects model. Statistical heterogeneity was evaluated using the Higgins I2 metric, with < 25 % as low, 25–75 % as moderate, and any value greater than 75 % as severe heterogeneity. We conducted a subgroup analysis by dividing patients into IBD subtypes: ulcerative colitis (UC) and Crohn’s disease (CD). To explore heterogeneity, we used the leave-one-out method by consecutively omitting one study at a time to identify the study contributing the most to heterogeneity. Publication bias was assessed by using funnel plots. Statistical significance for pooled effect estimate was considered if the 95 % CI did not cross the numeric value “1″ and the two-tailed p-value was less than 0.05.

## 3 Results

### 3.1 Study selection

A preliminary search using a predefined strategy yielded 529 records. After removing duplicates (n = 184), 345 records were subjected to preliminary screening by title and abstract assessment. We excluded 312 studies during the screening and subjected the remaining 33 studies to assessment of their respective full-texts. Subsequently, 26 articles were excluded during the full-text assessment for various reasons: ineligible outcomes (n = 15), no control group (n = 4), or different study design (case reports/series, letters, reviews) (n = 7). Finally, seven studies were included in the *meta*-analysis [18], [19], [20], [10], [11], [12], [13]. The study selection process is depicted in the PRISMA flowchart in Supplementary Figure S1.

### 3.2 Study and patient characteristics

A total of 88,893,407 patients were included, with 1,002,719 and 87, 890, 688 patients in the IBD and non-IBD groups, respectively. All seven included studies were observational in design. The publication years of the included studies ranged from 2014 to 2023.Mean ages ranged from 36.5 to 64.4 years and male to female ratio was comparable across the studies. Five of the included studies report about the presence of diabetes (2–16 %), hypertension (3–43 %), and dyslipidemia (4–20 %) in the IBD groups. The baseline and demographic characteristics of the studies are shown in Table 1.

**Table 1:** Baseline characteristics of included studies.

**Author Name (Year)**	**Study Design**	**Country**	**Number of patients; n**		**Inclusion Criteria**	**Age; “mean ± SD” OR “median (IQR)’**		**Males (%)**		**Diabetes Mellitus (%)**		**Hypertension (%)**		**Dyslipidemia (%)**		**Follow-up Duration (years); “mean ± SD” OR “median (IQR)”**	
			IBD	C		IBD	C	IBD	C	IBD	C	IBD	C	IBD	C	IBD	C
Baek 2016	Retrospective cohort	South Korea	3143	NA	Patients diagnosed with ARD and AF who were admitted to Severance Hospital between 2005–2015.	36.5 ± 17.1	NR	59.1	NR	4.7	NR	8.3	NR	4.8	NR	6.7 ± 4.4	
Choi 2019	Retrospective cohort	South Korea	37,696	113,088	Patients diagnosed with IBD between January 1, 2010 and December 31, 2014 from NHIS database.	39.4 ± 16.4	39.42 ± 16.4	61	61	3.9	4.7	10.9	12.1	6.7	7.0	4.9 ± 1.3	
Kristensen 2014	Retrospective cohort	Denmark	24,499	236,275	All Danish citizens aged 15 years or more at the time of initial diagnosis of CD or UC between 1 January 1996 and 31 December 2011 were included, if they within 1 year before or after diagnosis claimed a prescription for pharmacological IBD treatment.	43.9 ± 19.1	43.2 ± 18.7	46.1	45.9	2	0.8	3.3	1.3	NR	NR	6.8	6.9
Mubasher 2020	Retrospective cohort	United States of America	84 7,235	84,757, 349	Patients (≥18 years) diagnosed with IBD and cardiac arrhythmias (AF, atrial flutter, SVT, VT, VF) admitted between 2012 and 2014 from the AHRQ's NIS database.	52.45	57.41	43.09	40.9	16.23	25.29	31.40	39.10	20.22	29.53	NR	NR
Pattanshetty 2015	Retrospective cohort	United States of America	16	125	A diagnosis of UC or CD using EMR review between January 2001 and December 2010	64.4 ± 10.7	51.6 ± 15.7	NR	NR	12.5	8.1	43.8	24.6	NR	NR	7.9 ± 3.8	7.3 ± 3.5
Sun 2023	Retrospective cohort	Sweden	83,877	22,248	A diagnosis of IBD using ESPRESSO, a nationwide histopathology cohort, between 1969 and 2017 (using the SNOMED system)	NR											
Tilly 2023	Prospective cohort	United Kingdom	6128	494,072	Participants from the UK Biobank database were screened for rheumatic fever, gastrointestinal autoimmune diseases, musculoskeletal and connective tissue autoimmune diseases, and neurological autoimmune diseases, between 2006 and 2022.	NR										12.8 (1.6)	

Abbreviations: IBD: Inflammatory bowel disease; C: Control; ARD: Autoimmune rheumatic diseases; AF: atrial fibrillation; NHIS: National Health Insurance Service; UC: Ulcerative colitis; CD: Crohn’s disease; AHRQ: Agency for Healthcare Research and Quality; NIS: National Inpatient Sample; SVT: supraventricular tachycardia; VT: ventricular tachycardia; VF: ventricular fibrillation; EMR: Electronic medical records; ESPRESSO: Epidemiology Strengthened by histopathology Reports in Sweden; SNOMED: Systematized Nomenclature of Medicine; UK: United Kingdom and NR: Not reported.

### 3.3 Outcomes

Data comparing IBD and non-IBD groups were reported in six studies [18], [19], [10], [11], [12], [13]. IBD patients were at an increased risk of developing AF [RR: 1.52; 95 % CI: 1.19–1.95; p < 0.0009; I2:99 %] compared to the non-IBD group. No reduction in heterogeneity was observed in the sensitivity analysis.

**Fig. 1:**
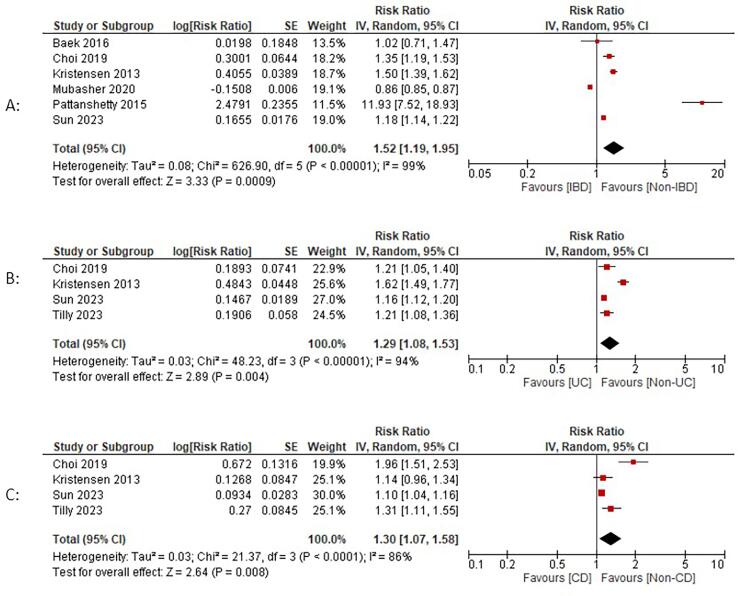
Individual and pooled analyses comparing atrial fibrillation (AF) risk in patients with inflammatory bowel disease (IBD). A: Inflammatory bowel disease; B: Ulcerative colitis subgroup; C: Crohn’s disease subgroup. Risk ratios (RR) with 95% confidence intervals (CI) are depicted on a logarithmic scale. The diamond symbolizes the combined or overall effect.

In subgroup analyses, UC patients were at an increased risk of developing AF [RR: 1.29; 95 % CI: 1.08–1.53; p = 0.004; I2 = 94 %] compared to the non-UC group. The removal of Kristensen et al. [11] reduced heterogeneity to zero. Similarly, CD patients also demonstrated an increased risk of AF development [RR: 1.30; 95 % CI: 1.07–1.58; p = 0.008; I2 = 86 %] compared to the non-CD group, and the removal of Choi et al. [10] reduced heterogeneity to 49 % (Fig. 1).

### 3.4 Quality assessment and publication bias

For quality appraisal of the included studies, we utilized NOS for observational studies. All studies were deemed “high quality” as depicted in Supplementary Table S3. Visual inspection of funnel plots was performed to assess possible publication bias, which demonstrated a low risk of bias (Supplementary Figure S2).

## 4 Discussion

Emerging research suggests a link between IBD and an increased likelihood of developing atherosclerotic cardiovascular diseases, heart failure, and AF [3]. This comprehensive *meta*-analysis delves into the intricate relationship between IBD and the development of AF.

Our findings indicate that individuals with IBD are at significantly increased risk of AF compared to those without the condition [RR: 1.52; 95 % CI: 1.19–1.95]. Among patients with IBD, those diagnosed with UC show a slightly higher risk of AF compared to those with CD. These results align with a previous *meta*-analysis by Zuin et al., which included three studies. Their analysis revealed an elevated risk of AF in IBD patients [OR: 2.26; 95 % CI: 2.11–2.41] [21]. A key strength of our study is the inclusion of larger number of studies compared to prior analyses. This expanded data set strengthens the generalizability and robustness of our conclusions.

Furthermore, our findings are supported by a prospective population-based UK Biobank (UKB) study, which demonstrated significant links between autoimmune diseases and the risk of developing new-onset AF. This study, encompassing around half a million participants, revealed substantial associations between new-onset AF and various autoimmune diseases, including rheumatic fever without heart involvement, gastrointestinal diseases (such as CD and UC), and musculoskeletal diseases (such as rheumatoid arthritis, psoriatic and enteropathic arthropathies, polyarteritis nodosa, systemic lupus erythematosus, and systemic sclerosis) [20]. Another longitudinal study by Kristensen et al. using Danish national healthcare records observed a similar increase in AF incidence specifically during periods of active IBD [11]. Interestingly, a study utilizing the U.S. National Inpatient Sample Database presented a contrasting perspective. This analysis found that IBD patients, typically younger, more often female, and with a higher smoking prevalence, exhibited lower rates of most arrhythmias compared to the general population. Notably, the exception was supraventricular tachycardia, which was more prevalent in the IBD group [19].

Chronic inflammation emerges as a pivotal factor linking IBD to AF [14]. Numerous studies have elucidated the intricate interplay between inflammation and cardiac electrophysiology, proposing mechanisms such as structural remodelling of atrial tissue, autonomic dysfunction, and oxidative stress. The chronic inflammatory state associated with IBD can lead to fibrosis and hypertrophy in the atrial tissue, creating a substrate conducive to the development and maintenance of AF. IBD has also been linked to autonomic nervous system imbalance, characterized by increased sympathetic activity and decreased parasympathetic tone, which can predispose individuals to AF by altering the electrical properties of the atria. Furthermore, elevated oxidative stress in IBD can cause direct damage to the atrial myocardium and promote AF by inducing inflammation and fibrosis [5], [15], [16].

**Fig. 2:**
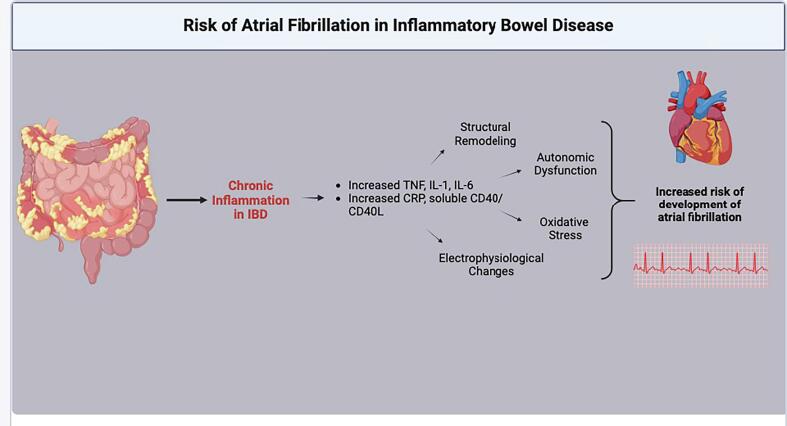
Schematic summarizing the cascade of events associating Inflammatory Bowel Disease (IBD) and Atrial Fibrillation (AF). *IBD: Inflammatory Bowel Disease; AF: Atrial fibrillation; TNF: Tumour Necrosis factor; IL: Interleukin; CRP: C-reactive protein; CD: Cluster of differentiation.

Further research corroborates our findings, further substantiating the heightened risk of AF in IBD patients. Notably, investigations into the molecular pathways underlying inflammation-induced arrhythmogenesis have underscored the role of pro-inflammatory cytokines, immune cell infiltration, and fibrosis in promoting atrial substrate remodelling conducive to AF development [5], [17]. Inflammatory molecules such as tumor necrosis factor, interleukin-1, and interleukin-6 which are characteristically elevated in IBD, have been shown to impact both the electrical and structural aspects of the heart, thereby increasing the risk of AF Moreover, elevated levels of the inflammatory marker C-reactive protein have been associated with a higher likelihood of developing or exacerbating AF. Some evidence also links the involvement of soluble CD40 and CD40L pathways, which are activated in patients with IBD, with the duration of non-valvular AF. These potential mechanisms underscore the complex interplay between inflammation, autonomic dysfunction, oxidative stress, and structural remodelling in the development of AF in IBD patients (Fig. 2). Further research is needed to elucidate the precise pathways and identify potential therapeutic targets to mitigate the increased risk of AF in this patient population.

This current *meta*-analysis underscores the clinical imperative of integrating AF screening into the holistic management of IBD patients. Recognizing AF as a potentially debilitating complication of IBD necessitates proactive surveillance strategies aimed at early detection and intervention. Leveraging insights from previous studies, clinicians can adopt tailored screening protocols, incorporating electrocardiographic monitoring and risk assessment tools to identify high-risk individuals promptly. By doing so, clinicians can mitigate the substantial burden of AF-related complications, including stroke, heart failure, and thromboembolic events, thereby optimizing patient outcomes.

### 4.1 Limitations

The results of this study must be interpreted with caution for several reasons. Firstly, the small number of total studies may be underpowered to detect statistical significance. Secondly, all of the included studies were observational. Observational studies are at an inherently higher risk of bias and they establish correlation but no causality. It is also crucial to note the lack of mechanistic insights in the included studies. Third, we observed high heterogeneity in all outcomes, which we attempted to address via sensitivity analyses. However, this could introduce variability and affect the reliability of the effect estimate. Lastly, this *meta*-analysis did not account for asymptomatic AF, thus underestimating the actual AF risk. Nonetheless, this current study highlights the clinical importance of integrating AF screening in IBD patients to mitigate poor outcomes.

### 4.2 Future directions

Future research should explore the impact of confounding variables such as disease severity, medication usage, and lifestyle factors on the observed association between IBD and AF. Using Mendelian randomization studies to analyse available genetic data (possibly from UKB) could help identify shared genetic loci associated with these conditions. It is also essential to investigate the age and gender distribution of AF cases and controls. Furthermore, it is important to determine if patients with IBD, who likely develop AF earlier than those without IBD, also have an earlier onset of AF compared to non-IBD controls. Longitudinal studies, augmented by advanced imaging modalities and biomarker assessments, offer promise in elucidating the temporal dynamics of inflammation-driven arrhythmogenesis and refining risk stratification strategies. Moreover, investigations into the therapeutic potential of anti-inflammatory agents, immunomodulatory therapies, and lifestyle interventions hold promise in attenuating the heightened risk of AF in IBD patients.

## 5 Conclusions

Patients with IBD are at higher risk of developing AF as compared to non-IBD individuals. Routine AF screening in IBD management is crucial to reduce the burden of AF-related complications in these patients. Multicenter, large-scale randomized trials are warranted to corroborate the results of this study.

## CRediT authorship contribution statement

**Aman Goyal:** Conceptualization, Data curation, Formal analysis, Methodology, Project administration, Software, Supervision, Writing – original draft, Writing – review & editing. **Hritvik Jain:** Conceptualization, Data curation, Investigation, Methodology, Resources, Supervision, Writing – original draft. **Surabhi Maheshwari:** Conceptualization, Data curation, Writing – original draft, Writing – review & editing. **Jyoti Jain:** Investigation, Methodology, Writing – original draft, Writing – review & editing. **Ramez M. Odat:** Supervision, Validation, Writing – original draft. **Humza Saeed:** Writing – original draft, Writing – review & editing. **Mohamed Daoud:** Conceptualization, Supervision, Validation, Visualization, Writing – review & editing. **Gauranga Mahalwar:** Validation, Writing – review & editing. **Kamna Bansal:** Conceptualization, Supervision, Validation, Visualization, Writing – review & editing.

## Declaration of competing interest

The authors declare that they have no known competing financial interests or personal relationships that could have appeared to influence the work reported in this paper.
