# Supplementary material for: Association between inflammatory bowel disease and atrial fibrillation: A systematic review and meta-analysis
Source: Int J Cardiol Heart Vasc. 2024 Jul 25;53:101456. doi: 10.1016/j.ijcha.2024.101456 (PMC11327605; doi:10.1016/j.ijcha.2024.101456)

**Supplementary Material**

**Association between Inflammatory Bowel Disease and Atrial Fibrillation: A Systematic Review and Meta-Analysis**

**Supplementary Table S1**. Search strategies for online databases.

**Supplementary Table S2**. Quality assessment using the Newcastle-Ottawa Scale.

**Supplementary Figure S1**. The Preferred Reporting Items for Systematic Reviews and Meta-Analyses (PRISMA) 2020 flowchart.

**Supplementary Figure S2.** Funnel plots for assessment of Publication Bias

This supplemental material has been provided by the authors to give readers additional information about their work.

**Supplementary Table S1**. Search strategies for online databases.

| Search Number | Database | Search Strategy | Number of Results |
| --- | --- | --- | --- |
| #1 | PubMed | ((inflammatory bowel disease) OR (ulcerative colitis) OR (crohns disease)) AND ((atrial fibrillation) OR (auricular fibrillation) OR (persistent atrial fibrillation) OR (paroxysmal atrial fibrillation)) | 81 |
| #2 | Embase | ((inflammatory bowel disease) OR (ulcerative colitis) OR (crohns disease)) AND ((atrial fibrillation) OR (auricular fibrillation) OR (persistent atrial fibrillation) OR (paroxysmal atrial fibrillation)) | 69 |
| #3 | Cochrane | ((MeSH descriptor: [Inflammatory Bowel Disease] explode all trees) OR (MeSH descriptor: [Colitis, Ulcerative] explode all trees) OR (MeSH descriptor: [Crohn Disease] explode all trees)) AND ((MeSH descriptor: [atrial fibrillation] explode all trees) OR ((auricular fibrillation) OR (persistent atrial fibrillation) OR (paroxysmal atrial fibrillation))) | 1 |
| #4 | Scopus | (TITLE-ABS-KEY(inflammatory bowel disease) OR TITLE-ABS-KEY(ulcerative colitis) OR TITLE-ABS-KEY(crohn disease)) AND (TITLE-ABS-KEY(atrial fibrillation) OR TITLE-ABS-KEY(auricular fibrillation) OR TITLE-ABS-KEY(persistent atrial fibrillation) OR TITLE-ABS-KEY(paroxysmal atrial fibrillation)) | 378 |
| TOTAL | | | 529 |

**Supplementary Table S2**. Quality assessment using the Newcastle-Ottawa Scale.

| **Study** | **Year** | **Selection** | | | | **Comparability** | **Outcome** | | | **Total Score** |
| --- | --- | --- | --- | --- | --- | --- | --- | --- | --- | --- |
|  |  | Representativeness of the exposed cohort | Selection of the non-exposed cohort | Ascertainment of exposure | Outcome of interest was not present at the start of the study |  | Assessment of outcome | Follow-up duration | Adequacy of follow-up |  |
| Baek | 2016 | ☆ | - | ☆ | ☆ | ☆ | ☆ | ☆ | ☆ | 7 |
| Choi | 2019 | ☆ | ☆ | ☆ | ☆ | ☆☆ | ☆ | ☆ | ☆ | 9 |
| Kristensen | 2013 | ☆ | ☆ | ☆ | ☆ | ☆ | ☆ | ☆ | ☆ | 8 |
| Mubasher | 2020 | ☆ | ☆ | ☆ | ☆ | ☆☆ | ☆ | - | - | 7 |
| Pattanshetty | 2015 | ☆ | ☆ | ☆ | ☆ | ☆ | ☆ | ☆ | ☆ | 8 |
| Sun | 2023 | ☆ | ☆ | ☆ | ☆ | ☆☆ | ☆ | ☆ | ☆ | 9 |
| Tilly | 2023 | ☆ | ☆ | ☆ | ☆ | ☆ | ☆ | ☆ | ☆ | 8 |

**Supplementary Figure S1**. The Preferred Reporting Items for Systematic Reviews and Meta-Analyses (PRISMA) 2020 flowchart.


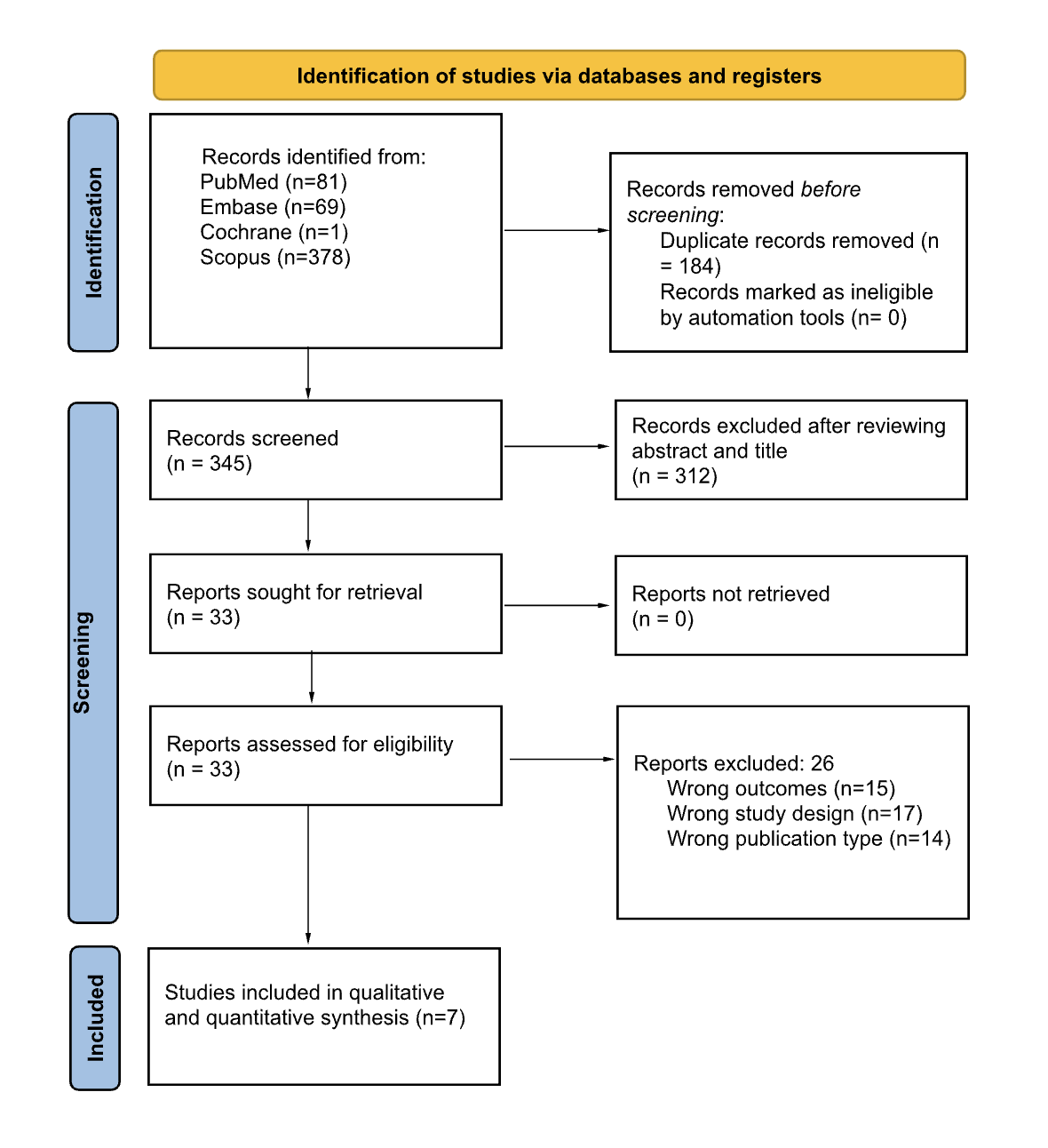


**Supplementary Figure S2**: Funnel plots for assessment of publication bias

A: Incidence of Atrial fibrillation in IBD vs Non-IBD group


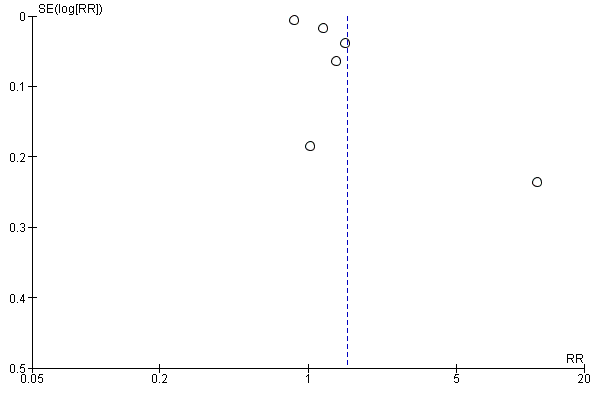


B: Incidence of Atrial fibrillation in patients with Ulcerative colitis


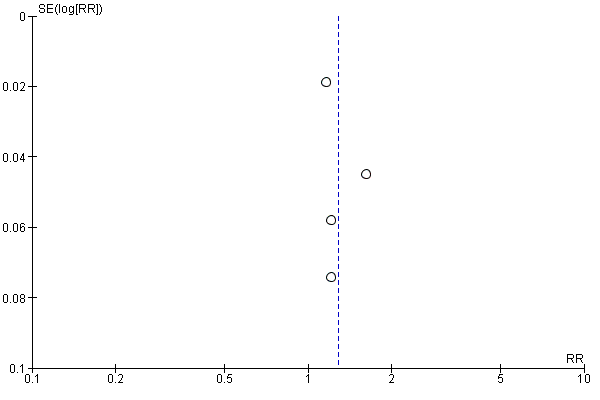


C: Incidence of Atrial fibrillation in patients with Crohn’s disease


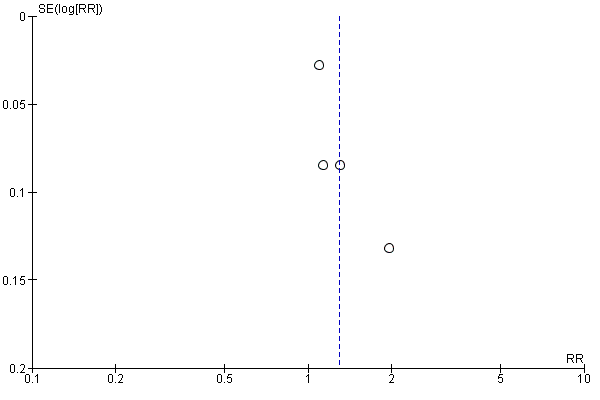

Supplement: Supplementary Data 1 [file mmc1.docx]
